# Factors Influencing on Pain in Patients Undergoing Pipelle Endometrial Biopsy for Abnormal Uterine Bleeding: Why a Personalized Approach Should Be Applied?

**DOI:** 10.3390/jpm12030431

**Published:** 2022-03-10

**Authors:** Milan Terzic, Gulzhanat Aimagambetova, Talshyn Ukybassova, Gauri Bapayeva, Aiym Kaiyrlykyzy, Faye Foster, Faina Linkov

**Affiliations:** 1 Department of Medicine, School of Medicine, Nazarbayev University, Zhanybek-Kerey Khans Street, 5/1, Nur-Sultan 010000, Kazakhstan; milan.terzic@nu.edu.kz (M.T.); faye.foster@nu.edu.kz (F.F.); 2 Clinical Academic Department of Women’s Health, CF “University Medical Center”, Turan Ave. 32, Nur-Sultan 010000, Kazakhstan; talshynu@yandex.ru (T.U.); gauri.bapayeva@gmail.com (G.B.); 3 Department of Obstetrics, Gynecology and Reproductive Sciences, University of Pittsburgh School of Medicine, 300 Halket Street, Pittsburgh, PA 15213, USA; 4 Department of Biomedical Sciences, School of Medicine, Nazarbayev University, Zhanybek-Kerey Khans Street, 5/1, Nur-Sultan 010000, Kazakhstan; 5 National Laboratory Astana, Center for Life Sciences, Nazarbayev University, Kabanbay Batyr Street, 53, Nur-Sultan 010000, Kazakhstan; aiym.kaiyrlykyzy@nu.edu.kz; 6 Health Administration and Public Health Department, Rangos School of Health Sciences, Duquesne University, 600 Forbes Ave., Pittsburgh, PA 15282, USA; faina.linkov@gmail.com

**Keywords:** abnormal uterine bleeding, Pipelle, endometrial biopsy, pain

## Abstract

Objectives. Abnormal uterine bleeding (AUB) is a common complaint of women in different age groups, and endometrial biopsy is widely used to investigate the underlying causes. The aim of this observational study was to assess factors influencing pain in patients undergoing endometrial biopsy for AUB. Methods. Pain intensity before, during, and after Pipelle sampling was evaluated using the numerical rating scale (NRS), where “0” represents no pain at all, “10”—the worst pain ever possible. Pain rating was categorized as 1–6—mild to moderate, 7 and above as severe pain. Results. The study included 160 women who underwent Pipelle biopsy. The median age in the cohort was 42 (34–48) years, 18.1% of women were postmenopausal, 56.3% were either overweight or obese, 30% were nulliparous and 80% reported urban residency. The median pain score during the procedure was 2 (0–4). Pain scores of 5 (4–7) were reported with the junior gynecologist and 2 (0–4) in the senior gynecologist (*p* < 0.0001). Conclusion. The pain was found to have a strong association with the type of provider performing the endometrial sampling procedure. This fact suggests the need for a personalized approach and that psychological or informational interventions should be scheduled before the procedure to decrease pain and increase satisfaction.

## 1. Introduction

Abnormal uterine bleeding (AUB) is a complaint of one-third of outpatient visits to gynecologist, more than two-thirds of consultations for peri- and postmenopausal patients, and 5% of emergency department visits [1,2,3,4]. Depending on the age, patients might have various underlying pathologies [5,6,7,8,9] and endometrial biopsy, such as conventional dilatation and curettage (D&C), hysteroscopy, and aspirations procedures, are used to establish the right diagnosis [1,3,10,11,12,13]. 

Conventional surgical procedures are performed under anesthesia, while office endometrial sampling without anesthetic still remains the challenge, as it might be linked to pain and discomfort [14], which in turn can impact the volume of endometrial tissue obtained during the procedure [15]. However, for a valid pathohistological assessment, there is a need to obtain a sufficient amount of the endometrial specimen [3,15,16]. Overall, there are many patient- and physician-related factors affecting the quality and amount of sample obtained [15,17]. In particular, the pain caused by the biopsy procedure can increase patient anxiety [18]. 

Impact of pain on the biopsy’s success remains underestimated and there are only a few reports of inconclusive results [14,19,20]. Considering that the relationship between Pipelle sampling and pain in patients undergoing endometrial biopsy for AUB has not been established yet, the aim of this study was to investigate factors influencing it. 

## 2. Materials and Methods

This is an observational study performed by analyzing the data of women who underwent endometrial biopsy. The patients’ recruitment took place from June 2019 to April 2021 at the Clinical Academic Department of Women’s Health of the University Medical Center (UMC), Nur-Sultan City, Kazakhstan. The study protocol was approved by the Institutional Research Ethics Committee of the Nazarbayev University (NU IREC) and the University Medical Center (25 February 2019). The guidelines outlined in the Declaration of Helsinki were followed. Each patient recruited to the study signed written informed consent.

The criteria/indications for endometrial biopsy included abnormal uterine bleeding in reproductive age, pre-, and postmenopausal patients. Therefore, the following inclusion criteria for the study were identified: (1) female; (2) age 18 and older; (3) endometrial biopsy recommended due to (but not limited to) abnormal uterine bleeding and irregular cycles (for pre-menopausal women) or post-menopausal bleeding. Exclusion criteria were the following: cervical cancer, pregnancy, acute pelvic inflammatory disease, blood coagulation disorders, acute cervical or vaginal infection, uterine anomalies/malformations, hysterectomy, previous endometrial ablation, or any intervention/procedure performed for Asherman’s syndrome. 

The Pipelle biopsy procedure was performed by two providers: junior specialist (less than 5 years of experience) and senior specialist with more than 35 years of experience as a gynecologist.

Demographic and clinical data were obtained from each participant during a medical interview. Pain intensity before, during, and after Pipelle sampling was evaluated using numerical rating scale (NRS) [21], where “0” represents no pain at all, “10”—the worst pain ever possible. Pain rating 1–6 was categorized as mild to moderate, 7 and above—as severe pain. 

Descriptive statistics were reported as median (IQR) for continuous variables and n (%) for categorical variables. Comparison of pain scores between patient’s menopausal status, body mass index, type of healthcare provider, parity, education, income, and residency was performed using Mann–Whitney U-test. Kruskal–Wallis test was utilized for testing pain differences between age groups and indication for current biopsy. Univariate and multivariate linear regression was run to understand the effects of type of healthcare provider on pain during biopsy performance. *p*-values < 0.05 were considered significant. All statistical analyses were performed with Stata 16 [22].

## 3. Results

The study included 160 women who underwent Pipelle biopsy, agreed to participate in the study and filled out the questionnaires. The median age in our cohort was 42 (34–48) years, 18.1% of women were postmenopausal, 56.3% were either overweight or obese, 30% were nulliparous and 80% reported urban residency (Table 1). 

We did not observe differences in the reported pain scores during biopsy performance between different age groups, menopausal status, biopsy indication, parity, body mass index (BMI), and socio-economic status such as residency, educational level, and income. However, women who underwent Pipelle biopsy with the junior gynecology specialist reported higher pain scores. The median pain score during the procedure was 2 (0–4) with the senior gynecology specialist, while women who underwent Pipelle sampling with the junior specialist reported median biopsy pain as 5 (4–7), (*p* < 0.0001).

## 4. Discussion

Abnormal uterine bleeding is very important in daily practice, and histologic assessment of endometrial tissue obtained with biopsy with exclusion of endometrial pathology as an underlying cause of it is essential [2,3,9,13,23]. The majority of endometrial sampling procedures are performed in outpatient settings, and the most important obstacle for the successful completion of procedures is pain [24]. Success rate of endometrial sampling refers to either the inability to reach uterine cavity or insufficiency of endometrial tissue obtained for histological analysis [12,15,25]. Pipelle endometrial biopsy is widely used for evaluation of AUB and performed in outpatient clinical settings without anesthesia. It has been found that multiple factors may have an impact on the biopsy results, including variable degree of pain during the procedure [14,15,16,26,27]. Conversely, in a recently published paper we revealed that pain had no influence on Pipelle success rate [28]. We proposed that proper patient information and understanding of Pipelle tool sampling benefits would facilitate a wide implementation of the technique and will have a positive impact on strategies for providing high-quality care for patients [28]. 

However, this is the first study to evaluate factors influencing pain in patients undergoing Pipelle endometrial biopsy for AUB. In this survey of 160 women, we found that women who underwent Pipelle biopsy performed by a junior gynecologist reported higher pain scores of 5 (4–7) in comparison with the same criteria evaluated during the intervention performed by the senior gynecologist—2 (0–4), (*p* < 0.0001). One of the rare recent studies on this topic reported severe pain in 9%, 43%, and 13% of participants prior to, during, and after the procedure, respectively [29]. 

One recently published study [14] compared pain scores among three office sampling methods (tampon, Tao brush, and endometrial biopsy using either Pipelle or EndoSampler) and identified the presence of pain during the procedure. Cited authors revealed that sampling using a tampon had significantly lower pain than both endometrial biopsy and Tao brush. Pain with tampon sampling was positively correlated with age and pain with endometrial sampling was inversely correlated with parity. Pain scores for Tao brush and endometrial biopsy were not significantly related to age, menopausal status, or BMI. Our study identified that the only factor influencing pain during Pipelle endometrial sampling was physician’s experience, while no statistical differences were observed in pain by age groups, menopausal status, biopsy indication, parity, BMI, residency, and educational level. Assuming the limited number of reports on this topic, it is essential to highlight that the physician’s experience impacts the pain level during the procedure, and this is the first study very precisely underlining this issue. 

In our previously published study, we reported the indications for endometrial biopsy and patient age being the factors that have an impact on biopsy success rates, both D&C and Pipelle. Moreover, the Pipelle biopsy was found to be highly reliable for the diagnosis of endometrial hyperplasia and endometrial carcinoma. Based on the results of that study, the endometrial sampling approach was recommended to be personalized and assessed individually, considering all of the factors influencing the reliability and success rates of the procedures [30]. Since the Pipelle biopsy is a cheap, simple to handle, safe, well tolerated, and a reliable office or outpatient tool, it can be the initial diagnostic method in the evaluation of AUB, except for patients with ultrasound scan results showing focal lesions such as endometrial polyps. The results of the study confirm the additional fact in the personalized approach to patients. Namely, we found that the only factor influencing pain during Pipelle endometrial sampling was the physician’s experience. For that, whenever the presence of factors decreasing the Pipelle success rate are realized, psychological or informational interventions before the procedure should be scheduled to decrease pain and increase patient satisfaction. Moreover, a senior physician with experience in Pipelle sampling should perform the procedure. 

Strengths and limitations. This is the first study in Kazakhstan related to Pipelle endometrial biopsy and factors influencing pain in patients undergoing this procedure. Moreover, this study is the first one identifying the importance of physicians’ experience on pain during Pipelle endometrial sampling. A small number of patients, missing data, and lack of association with other variables could be regarded as one of the limitations of this research. Another limitation of this research might be the fact that we analyzed the pain in patients undergoing Pipelle endometrial sampling performed by specialists in Obstetrics and Gynecology. In other countries and clinical settings worldwide, this procedure has been performed by General of Family Medicine Practitioners as well. Furthermore, the level of pain might be even higher if the procedure would have been performed by residents belonging to different years of these three different programs. Further large-scale studies with different psychological questionnaires and involving different specialties and residents might improve the results’ validity.

## 5. Conclusions

Pain was found to have a strong association with the type of provider performing the endometrial sampling procedure while association with other factors have not been found to be significant. The fact that pain is linked to the physician’s experience suggests that a personalized approach and psychological or informational interventions should be scheduled before the procedure for patients undergoing endometrial biopsy to decrease pain and increase satisfaction. 

## Figures and Tables

**Table 1:** Pain scores by patient characteristics.

	N(%)	Pain Score before Biopsy	*p*-Value	Pain Score during Biopsy	*p*-Value	Pain Score after Biopsy	*p*-value
Age group			0.2866		0.7676		0.16
≤44	94 (58.75%)	0 (0–2)		3 (1–5)		2 (1–4)	
45–54	47 (29.4%)	1 (0–2.5)		3 (1–5)		1 (1–3)	
≥55	19 (11.9%)	0 (0–2.5)		3 (1.5–5.5)		2 (0.5–4.5)	
Menopausal status			0.4755		0.8933		0.6017
Premenopausal	131 (81.9%)	0 (0–2)		3 (1–5)		2 (1–4)	
Postmenopausal	29 (18.1%)	0 (0–2)		3 (1–5)		2 (1–3)	
Body mass index			0.216		0.8147		0.9082
Normal	70 (43.7%)	0 (0–1.75)		3 (1–5)		2 (1–3)	
Overweight and obese	90 (56.3%)	1 (0–2)		3 (1–5)		2 (1–4)	
Type of provider			0.0087		<0.0001		0.15
Senior OBGYN	100 (62.5%)	1 (0–3)		2 (0–5)		2 (1–3)	
Junior OBGYN	60 (37.5%)	0 (0–1)		5 (3–6.25)		2.5 (1–4)	
Indication for biopsy			0.7038		0.6526		0.4174
Abnormal bleeding in reproductive age	97 (60.6%)	0 (0–2)		3 (1–5)		2 (1–4)	
Premenopausal bleeding	29 (18.1%)	1 (0–2)		3 (1.5–5.5)		1 (1–3)	
Postmenopausal bleeding	34 (21.3%)	0 (0–0.25)		3 (0.75–5)		1.5 (0.75–3.25)	
Parity			0.6513		0.3791		0.1083
None	48 (30%)	0 (0–2)		3 (0–5.25)		2 (1–4.25)	
One and more	112 (70%)	0 (0–2)		3 (1–5)		2 (1–3)	
Education			0.6854		0.6854		0.6309
None, technical and college	65 (40.6%)	0 (0–2)		3 (1–5)		2 (1–3)	
High, postgraduate	87 (54.4%)	0 (0–2)		3 (0.5–5)		2 (1–4)	
NA (missing values)	8 (5%)						
Income			0.6715		0.5148		0.2308
Not satisfactory	29 (18.1%)	1 (0–2)		3 (0–5)		1 (1–3)	
Satisfactory	91 (56.9%)	0 (1–0.25)		3 (1–5)		2 (1–4)	
NA (missing values)	40 (25%)						
Residency			0.7065		0.3226		0.08217
Urban	128 (80%)	0 (0–2)		3.5 (1–5)		2 (1–4)	
Rural	30 (18.8%)	1 (0–1.75)		3 (0.25–4.5)		1 (1–2)	
NA (missing values)	2 (1.2%)						

Data presented (Q1–Q3) for continuous and N (%) for categorical variables.

## Data Availability

The data from this study are available per reasonable request from the project PI, Milan Terzic, milan.terzic@nu.edu.kz.
